# Insulin Inertia Among People With Type 2 Diabetes Mellitus in Qatar: The INERT‐Q Study

**DOI:** 10.1002/edm2.495

**Published:** 2024-06-06

**Authors:** Mohammed Bashir, Noora Al Thani, Abeer Khalid, Obada Khalil, Zaina Alamer, Mohammed Khair Hamad, Gowri Karuppasamy, Mohammed Abufaeid, Mutwakil Elbidairi, Dhabia Al‐Mohnnadi, Tarik Elhadd, Mahmoud Zirie

**Affiliations:** ^1^ Endocrine Section, Internal Medicine Department Hamad Medical Corporation Doha Qatar; ^2^ Qatar Metabolic Institutes Hamad Medical Corporation Doha Qatar; ^3^ Department of Pharmacy Hamad Medical Corporation Doha Qatar

**Keywords:** insulin therapy, therapeutic inertia, Type 2 diabetes mellitus

## Abstract

**Background:**

Achieving and maintaining adequate glycaemic control is critical to reduce diabetes‐related complications. Therapeutic inertia is one of the leading causes of suboptimal glycaemic control.

**Aim:**

To assess the degree of inertia in insulin initiation and intensification in people with Type 2 diabetes mellitus (DM‐2).

**Methods:**

We performed a retrospective longitudinal cohort study and followed DM‐2 2 years before and 2 years after the start of insulin. The primary outcome was the proportion of patients who achieved glycaemic targets (HBA1c ≤ 7.5%) at 6th month, 1st year and 2nd year.

**Results:**

We included 374 predominantly male subjects (62%). The mean age was 55.3 ± 11.3 years, the mean duration of DM‐2 was 12.0 ± 7.3 years, 64.4% were obese, 47.6% had a microvascular disease, and 24.3% had a macrovascular disease. The mean HBA1c at −2nd year and −1st year was 9.2 ± 2.1% and 9.3 ± 2.0%, respectively. The mean HbA1C at the time of insulin initiation was 10.4 ± 2.1%. The mean HBA1c at 6th month, 12th month and 2nd year was 8.5 ± 1.8%, 8.4 ± 1.8% and 8.5 ± 1.7%, respectively. The proportion of subjects who achieved HBA1c targets at 6th month, 12th month and 2nd year was 32.9%, 31.0% and 32.9%, respectively. Multivariate logistic regression analysis showed that achieving HBA1c targets at 6th month and 1st year increases the odds of achieving HBA1c targets at 2nd year (OR 4.87 [2.4–9.6] *p* < 0.001) and (OR 6.2 [3.2–12.0], *p* < 0.001), respectively.

**Conclusion:**

In people with DM‐2, there was an alarming delay in starting and titrating insulin. The reduction in HBA1c plateaued at 6th month. Earlier initiation and intensification of insulin therapy are critical to achieving glycaemic targets. More studies are needed to examine the causes of therapeutic inertia from physicians', patients' and systems' points of view.

## Graphical abstract

- Type 2 diabetes mellitus (DM‐2) is a progressive disease, and many patients require insulin therapy, particularly those with early‐onset disease.
- Almost one‐third of the people with DM‐2 had evidence of complications at the time of insulin initiation.
- The higher the HBA1c at the time of insulin initiation, the lower the chances of achieving glycaemic targets.
- Delaying insulin titration beyond 6 months reduces the chances of achieving glycaemic targets at 2 years.
- Earlier insulin initiation and adequate titration are critical to achieving glycaemic targets in people with poorly controlled DM‐2.

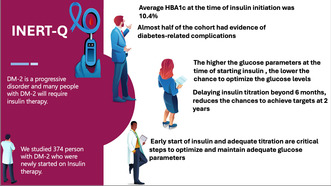

## 1 Introduction

Type 2 diabetes mellitus (DM‐2) is a chronic metabolic disorder affecting 536.6 million people worldwide with a projection increasing to 783.2 million in 2045 [1]. DM‐2 costs the global economy about 966 billion US$ per year, most of which is spent on managing diabetes‐related complications [1]. These diabetes‐related complications are a direct outcome of long‐term exposure to hyperglycaemia. Over the course of 20 years, continuous exposure to hyperglycaemia raises the chance of both myocardial infarction and death, according to recent data from the UKPDS [2]. The study also demonstrated that the risk of MI and death decreases with early HBA1c reduction. Thus, it is essential to reach and maintain glycaemic targets in patients with DM‐2 and reduce their exposure to hyperglycaemia.

DM‐2 is characterised by a progressive loss of beta‐cell secretory function, deriving the loss of glycaemic control over time, which requires regular medication intensifications [3]. Despite an adequate understanding of diabetes pathophysiology and its progressive nature, there is a global delay in medication intensification, particularly insulin. For patients with elevated HbA1C, a UK study with 80,000 patients with DM‐2 found a delay of 7 years before adding a second oral antidiabetic medication (OAD), 6.9 years before adding a third OAD agent and 6.1 years before adding a fourth OAD [4]. A multinational observational study for insulin initiation showed a consistent delay in insulin treatment as evident by a mean HBA1c of 8.9% at the time of enrolment [5]. A similar study from the Western‐Pacific region showed a mean HBA1c of 10.0% at the time of insulin initiation [6]. The delay in starting or intensifying medication is known as therapeutic Inertia. As outlined, the longer the exposure to high HbA1C, the higher the risk of complications [7]. Indeed, therapeutic inertia was shown to increase the rates of complication and the cost of managing DM‐2 [8, 9].

As a developing urban nation, Qatar has a high prevalence of obesity and DM‐2 (17%) [10]. In addition, DM‐2 is highly prevalent (11%) in individuals under 45 years in Qatar [10]. Thereby, the earlier age of onset of DM‐2 in Qatar implies a more prolonged exposure to hyperglycaemia, resulting in earlier and higher risks of complications if inadequately managed. Furthermore, the onset of diabetes at a younger age is associated with accelerated loss in beta‐cell function, and hence, patients with DM‐2 in Qatar are likely to require insulin earlier and at a younger age [11]. Hence, studying and addressing therapeutic inertia in this high‐risk population is critical. This study examines the degree of insulin inertia in routine clinical practice in Qatar.

## 2 Methods

### 2.1 Study Design and Population

We undertook a retrospective observational longitudinal study of patients with DM‐2 who attended Hamad General Hospital, a tertiary teaching and research centre in Doha, Qatar. We included those ≥18 years who were first started on insulin between June 2016 and June 2018, had biochemical data for at least 2 years before the start of insulin and attended at least one visit after insulin was started. We excluded patients with positive anti‐GAD antibodies. On average, patients with DM‐2 are seen once every 6 months.

**FIGURE 1:**
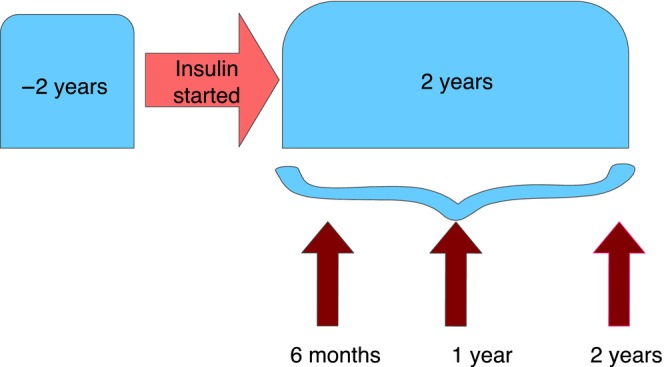
The study time points.

The primary outcome was the proportion of patients achieving HBA1c targets (≤7.5%) at 6th month. Secondary outcomes included the HBA1c threshold for starting insulin therapy, the average duration of diabetes at the start of insulin therapy, the median number of OAD at the start of insulin, the proportion of patients achieving targets at 1st and 2nd years; changes in average HBA1c over time; changes in total daily insulin doses over time; changes in BMI, blood pressure and lipids profile; changes in estimated glomerular filtration rate (eGFR) and albuminuria; the proportion of patients with new‐onset retinopathy, nephropathy, neuropathy and cardiovascular disease. Figure 1 shows the study time points. Approximately 9000 patients were screened, and 374 patients met the inclusion criteria.

### 2.2 Data Collection and Definitions

Data were collected using the pharmacy database to identify patients who started newly on Insulin from 01 June 2016 to 01 June 2018. We used electronic medical records—Cerner to collect the rest of the data. Cerner is the national medical records used by both primary and secondary health care in Qatar. At baseline, we collected the following information: age, gender, nationality, weight, height, BMI, age of onset of DM‐2, duration of DM‐2, HbA1c, renal function, liver function, fasting lipids, urine albumin creatinine ratio (ACR), other antidiabetic medications, duration of treatment on other antidiabetic medications and diabetes‐related complications. Over the follow‐up period, data about insulin intensification, changes in other medication, BMI, HbA1C, renal and liver function, fasting lipid profile and urine ACR will be recorded at 6th month, 12th month and 24th month. Further information about the prevalence of micro‐ and macrovascular complications was included at baseline and end of the study.

The glycaemic target was defined as target HbA1C ≤ 7.5%. Treatment intensification is defined as any changes in insulin doses or the addition of new antidiabetic medications in patients with HBA1c > 7.5%. eGFR was calculated using the CKP‐EPI formula [12]. We used ethnic‐specific cut‐off points to define normal weight, overweight and obesity [13].

We selected this HBA1c as opposed to the classical HBA1c target of ≤7.0% for a few reasons. Most randomised controlled trials that examined insulin initiation in people with DM‐2 achieved a mean HBA1c between 7.2 and 7.5%, with only one‐third of the subjects achieving that target HBA1c of ≤7.0% [12, 13]. These trials have extensive resources deployed for close glucose monitoring and insulin adjustment. Furthermore, several studies have shown that the risk of both severe hypoglycaemia and death is increased with intensive therapy in people with DM‐2 [14, 15, 16]. Hence, we considered a target HBA1c of ≤7.5% in people with DM‐2 on insulin to be both practical and safe.

### 2.3 Statistical Analysis

We used STATA 15 for statistical analysis. Continuous data are summarised as mean (S.D.) and median (IQR) as appropriate, although discrete data are summarised as percentages. Continuous data were compared at each time point with baseline using paired *t*‐tests. Categorical data were compared using the chi‐squared test. We performed univariate and multivariate regression analysis for the predictors of HBA1c at 6th month. We incorporated variables with *p* value ≤ 0.1 in the univariate analysis.

Similarly, we performed univariate and multivariate logistic regression analysis for factors that predict achieving glycaemic targets at 2nd year. In the multivariate regression model, we incorporated variables with *p* value ≤ 0.10 in the univariate analysis. We considered *p* value < 0.05 to be statistically significant.

## 3 Results

We included 374 patients in the final analysis (Table 1). Most of the patients were males (62%), the mean age was 55.3 ± 11.3 years, the mean duration of DM‐2 was 12.0 ± 7.3 years, the mean age of onset of DM‐2 was 41.2 ± 9.6 years, and 64.4% were obese. The mean HbA1C at the time of insulin initiation was 10.4 ± 2.1%, whereas it was 9.2% and 9.3% in the preceding 2 years. Most patients were on 2–3 oral agents, 38.8% and 37.4%, respectively. Table 2 shows that 24.3% of the patients had established macrovascular disease, and 47.6% had microvascular. Diabetic nephropathy was the most common microvascular complication (32.1%), followed by diabetic retinopathy (18.7%) and neuropathy (16.3%).

**TABLE 1:** Baseline characteristics.

Gender	
Males	232 (62.0%)
Females	142 (38.0%)
Ethnicity	
Qatari	130 (34.8%)
Arab	90 (24.1%)
Asian	143 (38.2%)
Others	11 (2.9%)
Age (years)^a^	55.3 years ±11.3
Age of DM‐2 onset (years)^a^	41.2 ± 9.6
Duration (years)^a^	12.0 ± 7.3
Weight (kg)^a^	81.8 ± 17.6
BMI (kg/m^2^)^a^	30.6 ± 6.6
Obesity^b^	
Normal Weight	43 (11.9%)
Overweight	86 (23.8%)
Obese	233 (64.4%)
Systolic BP (mmHg)^a^	133 ± 18
Diastolic BP (mmHg)^a^	76 ± 9.8
HBA1c (%)^a^	10.4 ± 2.1
Total cholesterol (mmol/L)^a^	4.5 ± 1.4
LDL (mmol/L)^a^	2.5 ± 1.0
HDL (mmol/L)^a^	1.05 ± 0.3
Trig (mmol/L)^a^	2.3 ± 3.2
Creatinine (umol/L)^a^	84.3 ± 41
eGFR^a^	91.7 ± 24
Metformin^b^	302 (80.7%)
DPP4 I^b^	243 (65.0%)
SU^b^	200 (53.5%)
SGLT2i^b^	32 (8.6%)
TZDs^b^	30 (8.0%)
GLP‐1RA^b^	35 (9.4%)
No. of OHA^b^	
One	63 (18.3%)
Two	134 (38.8%)
Three	129 (37.4%)
Four	19 (5.5%)

^a^ Mean ± Standard deviation.

^b^ Number (%).

**TABLE 2:** Diabetes‐related vascular complications rates at baseline.

Composite microvascular complications	178 (47.6%)
Retinopathy	70 (18.7%)
Retinopathy type	
NPDR	49 (13.1%)
PDR	21 (5.6%)
Neuropathy	61 (16.3%)
Painful neuropathy	36 (9.6%)
Albuminuria	
Normal	73 (41.0%)
Microalbuminuria	75 (42.1%)
Macroalbuminuria	30 (16.9%)
Diabetic nephropathy	198 (32.1%)
DKD stages	
Stage 1	233 (62.8%)
Stage 2	91 (24.5%)
Stage 3 a	23 (6.2%)
Stage 3 b	18 (4.9%)
Stage 4	5 (1.3%)
Stage 5	1 (0.3%)
Composite macrovascular complications	91 (25.3%)
Cardiovascular disease	76 (20.3%)
Cerebrovascular disease	17 (4.6%)

Most patients were started on basal insulin 68.7%, with a mean insulin dose of 0.21 ± 0.11 unit/kg/day, whereas only 4.8% were started on premixed insulin with a mean insulin dose of 0.5 ± 0.3 units/kg/day, as demonstrated in Table 3.

**TABLE 3:** Types of insulin and doses started.

Insulin type	
Basal	257 (68.7%)
MDI	98 (26.2%
Mixed insulin	18 (4.8%)
Bolus	1 (0.3%)
Median insulin starting doses (units)	
Basal	15 (10–20)
MDI	38 (28–57)
Mixed	30 (24–50)
Bolus	20
Mean insulin dose per kg (units/kg)	
Basal	0.21 ± 0.11
MDI	0.53 ± 0.25
Mixed	0.5 ± 0.3
Bolus	0.27

### 3.1 Glycaemic Control

**FIGURE 2:**
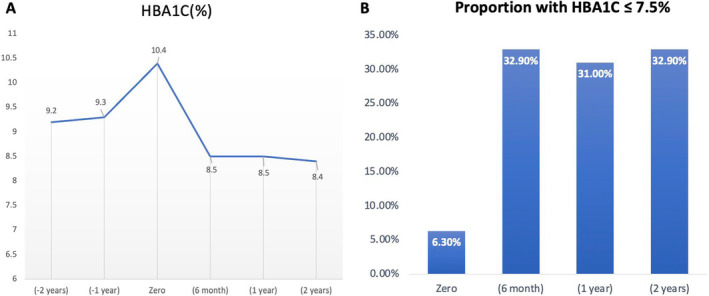
(A) Changes in mean HBA1c over the study period. (B) The proportion of patients with HBA1c ≤ 7.5%.

**FIGURE 3:**
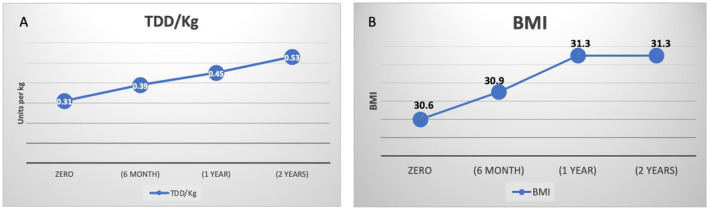
(A) Changes in the total daily dose of insulin per kg. (B) The changes in mean BMI.

We had glycaemic data on 322, 342 and 356 subjects at 6th month, 12th month and 2nd year, respectively. The mean HBA1c at 6th month, 12th month and 2nd year was 8.5 ± 1.8%, 8.4 ± 1.8% and 8.5 ± 1.7%, respectively (Figure 2A). The proportion of patients who achieved targets at 6th month, 12th month and 2nd year were 32.9%, 31.0% and 32.9%, respectively (Figure 2B). Furthermore, there was a steady rise in the average BMI and the total daily doses of insulin/kg (Figure 3). As shown in Table S1, at 6th month, there were no differences in baseline data or insulin doses between those who did and did not achieve HBA1c targets.

According to the multivariate regression analysis (Table 4), the HBA1c levels at 6th month increase by 0.05% (*p* = 0.023), 0.17% (*p* = 0.017) and 0.19% (*p* = 0.011) for every unit increase in BMI, HBA1c at −1st year and HBA1c at the start of insulin, respectively. For every 1‐year increase in the age of DM‐2 onset, HBA1c at 6th month decreased by 0.03% (*p* = 0.013). Multivariate logistic regression analysis showed that the odds of achieving HBA1c targets at 2nd year were higher among those who achieved targets at 6th month (OR 4.87 (2.4–9.6) *p* < 0.001) and at 1st year (OR 6.2 (3.2–12.0), *p* < 0.001), whereas it was lower in those with established macrovascular disease (OR 0.38 (0.16–0.89) *p* = 0.027) (Table 5).

**TABLE 4:** Multivariate regression analysis for HBA1c at 6th month.

	β coefficient (95% CI)	95% CI
		*p*
HBA1c (−1 year)	0.17 (0.04–0.34)	0.011
HBA1c—at the initiation of insulin	0.2 (0.03–0.31)	0.017
Age of onset	−0.03 (−0.05 to −0.01)	0.013
BMI	0.05 (0.01–0.09)	0.023
Total daily dose	−0.012 (−0.03 to 0.004)	0.162
Mixed insulin	0	
Basal alone	−0.63 (−2.0 to 0.78)	0.380
MDI	−0.58 (−2.0 to 0.87)	0.431

**TABLE 5:** Multivariate logistic regression analysis for HBA1c targets at 2nd year.

	OR (95% CI)	*p*
Achieving targets at 6th month	4.87 (2.4–9.6)	<0.001
Achieving targets at 1st year	6.2 (3.2–12.0)	<0.001
Macrovascular complications	0.38 (0.16–0.89)	0.027

## 4 Discussion

This retrospective longitudinal study of 374 patients showed a minimum of 2 years of delays in starting Insulin in patients with uncontrolled DM‐2. By the time insulin was started, 47.6% and 24.3% of the patients had established microvascular and macrovascular complications, respectively. After the start of insulin, there was a rapid reduction in the average HBA1c by 1.9% in the first 6 months, and only one in three patients achieved glycaemic targets. Furthermore, the study showed that delaying insulin is associated with lower chances of achieving glycaemic targets at 6th month. It also showed that if the glycaemic targets are not achieved at 6th month, it is unlikely to improve. Furthermore, the higher the age of DM‐2 onset, the better the response to treatment.

A critical finding of this study is that the demographics of DM‐2 patients who are starting insulin in Qatar are different from other populations. The mean age of DM‐2 onset (41 years) is much younger, whereas the mean duration of DM‐2 (12 years) and the mean HBA1c at the time of insulin therapy (10.4%) are much higher than other populations. A cohort study of patients with DM‐2 starting on insulin from 12 countries showed that the average DM‐2 onset was 51 years, the average duration of DM‐2 was 9.8 years, and the mean HBA1c was 8.9% [5]. Another large cohort study of new starters on insulin reported a mean age of DM‐2 onset of 58.2 years, a mean DM‐2 duration of 8.5 years, and a mean HBA1c of 9.7% [14]. This young age of onset is a significant concern. Compared to those without diabetes, early‐onset DM‐2 is associated with 7–11 years of life lost [15].

Furthermore, early‐onset DM‐2 is associated with an accelerated loss of beta‐cell function and a higher proportion of micro‐ and macrovascular complications [15]. Hence, the combination of the younger age of DM‐2 onset and the more prolonged exposure to hyperglycaemia is expected to produce high rates of complications. Indeed, the prevalence of established microvascular complications (47.6%) and macrovascular complications (24.3%) is much higher than in other studies. The multinational study reported that 33% and 27% had established microvascular and macrovascular complications [5]. A study from six Western countries reported that 16.7% and 14.2% had established microvascular and macrovascular complications when insulin was started [16]. However, a study that included 28 Asian countries reported similarly high levels of complications; 53.3% had microvascular complications, and 27.2% had macrovascular complications [17].

Another critical finding is that early insulin intensification is central to achieving glycaemic targets. At 6th month, the mean HbA1c was reduced by 1.9%, with no further improvement. Patients who achieve glycaemic targets at 6th month are likelier to maintain this by 2 years. These results support the findings from other studies. Mauricio et al. reported a similar pattern plateau in the HBA1c reduction at 6th month in 40,000 patients with DM‐2 from six Western countries who were newly started on insulin [16]. The study also showed that those who did not achieve HBA1c targets (≤7.0%) at 3rd month were unlikely to do so in 2 years' time [16]. Furthermore, the suboptimal increase in the total daily dose of insulin (TDD/kg) from 0.31 to 0.53 units/kg suggests inertia in insulin titration. Clinical trials showed that most patients with DM‐2 on insulin require a TDD between 0.7 and 1.3 U/kg to achieve glycaemic targets [18, 19]. This suboptimal escalation in insulin doses was reported in other studies. A study from Japan reported an increase in the TDD from 0.16 U/kg to 0.24 U/kg at the end of 18 months, with most patients not achieving glycaemic targets [20]. Another study from Brazil showed that the TDD of insulin ranged between 0.24 U/kg and 0.39 U/kg by 2 years in patients with DM‐2 who were newly started on insulin [21].

Therapeutic inertia is not uncommon. A recent meta‐analysis of 25 studies showed that therapeutic inertia affects 50% of patients with DM‐2 [22]. Excess glycaemic exposure increases the risk of complications. Paul et al. showed that for every 1‐year delay in reducing hyperglycaemia, the risk of myocardial infarction, stroke and heart failure increased by 67%, 51% and 64%, respectively [23]. Insulin remains a critical therapeutic intervention, and the newer armamentarium had minimal effects on the timing of insulin. A German study followed two cohorts with newly diagnosed DM‐2 before and after the broader use of SGLT2i and GLP1‐RA and showed that the mean time to starting insulin has increased by only 200 days [24]. Unfortunately, the delay in starting insulin is not uncommon [5, 25, 26]. Therapeutic inertia is multifactorial, with factors related to the physicians, patients and systems [27]. Realising its impact on patient care, the American diabetes association published an initiative to overcome therapeutic inertia [28]. A key recommendation is to quantify the magnitude of therapeutic inertia and then examine the causes before building strategies to overcome it.

Being retrospective is the main limitation of this study. We had no data on hypoglycaemia—a significant barrier to insulin intensification. We also did not collect data on medication intensification in 2 years prior to the study. The study's main strength is the strict protocol that allowed us to collect comprehensive data.

## 5 Conclusion

In this study of patients with DM‐2 who attended a large secondary care centre in Qatar, there was a concerning delay in starting and titrating insulin. The combination of the younger age of onset of DM‐2 and therapeutic inertia is feared to increase the prevalence of diabetes‐related complications. Indeed, almost one in two of the patients had either microvascular or macrovascular complications. More studies are needed to examine the causes of therapeutic inertia from physicians', patients' and systems' points of view.

## Author Contributions

M.B. has contributed to the conceptualisation, design, data analysis and interpretation of the study and has written the manuscript. N.A.T. has contributed to the conceptualisation and study design, supervised data collection and data validation and has co‐written the manuscript. A.K., O.K., Z.A., M.K.H., G.K., M.A. and M.E. have collected and validated the data and reviewed the manuscript. D.A., T.E. and M.Z. have contributed to the study design data interpretation and reviewed the manuscript. All authors approved the final manuscript as submitted and agreed to be accountable for all aspects of the work. All authors declare that the work is original and has not been submitted or published elsewhere.

## Ethical Statement

The Internal Review Board (IRB) of Hamad Medical Corporation approved the study—MRC‐01‐21‐197. Because of the retrospective nature of the study, the IRB waived the consent form.

## Conflicts of Interest

The authors declare no conflicts of interest.

## Supporting information


Table S1

## Data Availability

The data that support the findings of this study are available from the corresponding author upon reasonable request.
