# Supplementary material for: Insulin Inertia Among People With Type 2 Diabetes Mellitus in Qatar: The INERT‐Q Study
Source: Endocrinol Diabetes Metab. 2024 Jun 6;7(4):e00495. doi: 10.1002/edm2.495 (PMC11156521; doi:10.1002/edm2.495)
Supplement: Supplementary file 1 — Table S1 [file EDM2-7-e00495-s001.docx]

Supplementary table 1

| Six-month outcomes | | | ≤ 7.5% (106) | >7.5% (216) | P Value |
| --- | --- | --- | --- | --- | --- |
| Age (years) | | | 54.9± 11.9 | 55.6 ± 11.2 | 0.605 |
| Duration | | | 11.± 7.4 | 12.4 ± 7.1 | 0.1635 |
| Age of onset | | | 42 ± 9.5 | 41.2 ± 9.7 | 0.5003 |
| Males (%) | | | 71 (67.0%) | 130 (60.2%) | 0.237 |
| BMI | | | 28.6 ± 5.6 | 30.6± 6.7 | 0.1715 |
| Ethnicity | | Qatari | 33.0% | 35.2% | 0.916 |
|  |  | Arab | 21.7% | 23.2% |  |
|  |  | Asian | 41.5% | 38.9% |  |
|  |  | Other | 3.7% | 2.85 |  |
| Insulin type | Basal | | 67.9% | 68.5% | 0.255 |
|  | MDI | | 30.2% | 26.4% |  |
|  | Mixed | | 1.9% | 5.1% |  |
| Total daily doses/kg Units | | | 0.3 | 0.3 | 0.3065 |
| OHA (mean) | | | 2.2 | 2.3 | 0.458 |
